# (−)-Epicatechin regulates endoplasmic reticulum stress and promotes ferroptosis in lung cancer cells via the PERK/eIF2α/ATF4 signaling pathway

**DOI:** 10.1371/journal.pone.0313010

**Published:** 2024-10-31

**Authors:** Zengbo Lv, Peiwan Liu, Yingyu Yang, Jianhua Ji, Anao Wu, Wensheng Huang, Liqiong Zhang, Zhijun Zhang, Yunkui Yang, Wenhui Li, Meifang Huang

**Affiliations:** 1 Department of Oncology, The First People’s Hospital of Qujing/The Qujing Affiliated Hospital of Kunming Medical University, Qujing, Yunnan, China; 2 Department of Hepatobiliary Surgery, The First People’s Hospital of Qujing/The Qujing Affiliated Hospital of Kunming Medical University, Qujing, Yunnan, China; 3 Department of Pathology, The First People’s Hospital of Qujing/The Qujing Affiliated Hospital of Kunming Medical University, Qujing, Yunnan, China; 4 Department of Radiotherapy, The Third Affiliated Hospital of Kunming Medical University, Kunming, Yunnan, China; 5 Geriatrics Department, The First People’s Hospital of Qujing/The Qujing Affiliated Hospital of Kunming Medical University, Qujing, Yunnan, China; 6 Department of Oncology, The Third Affiliated Hospital of Kunming Medical University, Kunming, Yunnan, China; BRAC University, BANGLADESH

## Abstract

**Objective:**

(−)-Epicatechin (EC) is an active ingredient of *Fagopyrum dibtrys* (D. Don) Hara and can regulate lung cancer progression. However, the specific regulatory mechanism is poorly understood. This study explored the specific mechanism of EC in the treatment of lung cancer.

**Methods:**

H460 cells were injected subcutaneously into the left dorsal sides of nude mice to establish an animal model of lung cancer. H460 and H1299 cells and nude mice were treated with different concentrations of EC. The expression levels of related proteins were detected by Western blotting. Cell proliferation, migration, and invasion were detected by CCK-8, colony formation, and Transwell assays. Flow cytometry was used to detect the Ca^2+^ level in lung cancer cells. Immunohistochemistry was used to detect the expression of Ki-67 in tumor tissues.

**Results:**

This study revealed that ferroptosis in lung cancer cells was inhibited during lung cancer development. EC treatment promotes ferroptosis, inhibits the proliferation, migration and invasion of lung cancer cells, and inhibits the formation of tumors in vivo. Ferroptosis inhibitors (Fer-1) weaken the effects of EC on lung cancer cells, whereas a ferroptosis inducer (erastin) further promotes the effects of EC. In addition, endoplasmic reticulum (ER) stress is involved in the EC-induced ferroptosis of lung cancer cells, and treatment with GSK, an inhibitor of the ER stress protein PERK, can reverse the effect of EC.

**Conclusion:**

EC therapy activates the PERK–eIF2α–ATF4 signaling pathway to increase ER stress, thereby promoting ferroptosis in lung cancer cells and inhibiting the occurrence and development of lung cancer. Our research suggests that EC may become a drug candidate for treating lung cancer.

## 1 Introduction

Lung cancer is one of the most common malignant tumors in the world, causing 1.6 million deaths every year [1]. Its incidence and mortality rates are increasing annually, and it now ranks first among malignant tumors that cause death [2]. Smoking, exposure to second-hand smoke, air pollution, occupational exposure (such as asbestos and radiation), and familial inheritance are the main risk factors leading to the occurrence of lung cancer [3]. Owing to the lack of obvious symptoms in early-stage lung cancer, the disease is difficult to detect, resulting in most patients having an advanced stage by the time of diagnosis [4]. Therefore, comprehensive treatment based on radiotherapy and chemotherapy has become the main treatment method for patients with advanced lung cancer [5], but its efficacy is limited, and these patients are prone to radiation resistance and recurrence. Therefore, active explorations for drug candidates to treat lung cancer should be undertaken to improve the tumor local control rate.

The low toxicity and high efficiency of traditional Chinese medicine have promoted its development. *Fagopyrum dibtrys* (D. Don) Hara is a perennial herb of the Polygonaceae family that is used as a medicine with rhizomes, has the effect of "clearing heat and detoxifying and draining pus and removing stasis", and was included in the 2015 edition of the Chinese Pharmacopoeia [6]. The active ingredient (−)-epicatechin (EC) of *Fagopyrum dibtrys* (D. Don) Hara has been reported to have potential antitumor effects [7]. For example, Takanashi [8] reported that EC can inhibit the proliferation and migration of PC-3 prostate cancer cells, induce cell cycle G2 phase arrest, and alleviate the development of prostate cancer. In addition, Saha et al. [9] reported that EC inhibits the growth of PC-9 and A549 lung cancer cells and promotes their apoptosis, but its mechanism of action has not been thoroughly studied. This study explored the specific mechanisms by which EC regulates the progression of lung cancer.

With the continuous deepening of research on lung cancer, many studies have shown that targeted ferroptosis of lung cancer cells may be a new method for treating the disease [10]. Ferroptosis is an iron-dependent form of cell death characterized by the accumulation of intracellular iron and increases in the levels of reactive oxygen species and lipid peroxidation [11]. Research has shown that promoting ferroptosis can inhibit the growth and migration of lung cancer cells [12]. More importantly, studies have shown that EC can affect the process of ferroptosis by regulating the expression levels of genes related to ferroptosis [13]. However, there are currently no reports demonstrating that EC affects lung cancer progression by regulating ferroptosis.

In recent years, research has shown that ferroptosis in cells is often accompanied by a stress response in the endoplasmic reticulum [14]. The endoplasmic reticulum (ER) is a dynamic organelle that coordinates the folding and posttranslational maturation of almost all membrane proteins and most secretory proteins. When cells are subjected to external stress stimuli, such as oxidative stress and drug stress, ER function may be damaged, leading to abnormal protein synthesis and folding, thereby triggering ER stress [15]. A recent study revealed that ER stress can induce ferroptosis in cells [16]. In addition, studies have shown that the occurrence of ferroptosis is accompanied by PERK/eIF2α/activation of the ATF4 signaling pathway [17]. The PERK/eIF2α/ATF4 signaling pathway is an important signaling pathway that induces ER stress [18]. When ER stress is triggered, PERK is activated and phosphorylated, thereby phosphorylating eIF2α [19]. However, this also results in preferential translation of ATF4 mRNA, leading to an increase in its expression [20]. Therefore, exploring the PERK/eIF2α/ATF4 axis may contribute to the development of new lung cancer diagnosis and treatment strategies by influencing ER stress to regulate ferroptosis in cancer cells.

In summary, this study aims to explore in depth the role of EC in the PERK/eIF2α/ATF4 signaling pathway, which regulates the effect of ER stress on ferroptosis in lung cancer cells. The study also aims to clarify its mechanism of action and provide a theoretical basis for the development of new treatment methods for lung cancer.

## 2 Materials and methods

### 2.1 Cell culture

The human normal pulmonary bronchial epithelial cell line BEAS-2B and the human lung cancer cell lines H460 and H1299 were obtained from Shenzhen Otwo Biotechnology Co., Ltd. BEAS-2B cells were cultured in DMEM (HyClone), and H460 and H1299 cells were cultured in RPMI-1640 medium (Gibco, USA). The media were supplemented with 10% fetal bovine serum, 100 U/mL penicillin, and 100 mg/mL streptomycin, and the cells were placed in a culture incubator at 37 °C and 5% CO_2_ for routine cultivation. Then, 0, 5, 10, 20, or 40 μg/mL EC (Solarbio, IE0120) was added to the cultured H460 and H1299 cells for 24 hours, and the IC50 concentrations in the cancer cells were calculated. BEAS-2B, H460, and H1299 cells were cultured at these concentrations for 24 hours for subsequent experiments. For the ferroptosis inhibitor (Fer-1) group, 10 μmol/L Fer-1 (Sigma–Aldrich) was added; for the ferroptosis activator (erastin) group, 5 μM erastin (Sigma–Aldrich) was added; and for the PERK inhibitor (GSK) group, 15 μg/mL GSK (Sigma–Aldrich) was added.

### 2.2 Animal model construction

Forty 6-week-old male BALB/c nude mice (weighing 16–18 g) were obtained from the Animal Experimental Center of Kunming Medical University for research. Before the start of this study, all researchers involved in the nude mouse experimental operations received special training, acquired the animal -care and—handling capabilities required for nude mouse experiments, and were able to carry out this study smoothly under the premise of ensuring animal welfare. After one week of adaptive feeding, the experimental animals were randomly divided into four groups with 10 nude mice in each group. H460 cells (1×10^5^) were subcutaneously injected into the left dorsal side of each nude mouse. Starting from the 0th day after cell injection, ECs were used for treatment. The EC treatment group was administered 25, 50, or 100 mg/kg/d EC orally for 15 days. The course of treatment for EC in the study was set at 15 days based on our previous pretrial results. A 15-day course of treatment was able to fully demonstrate the efficacy of EC but also prevent the possible side effects of long-term treatment. The health and behavior of the nude mice were monitored daily. The painkiller buprenorphine (0.1 mg/kg) was injected into the animals that presented pain symptoms to reduce any pain caused by the experiment. After 28 days of cell injection, the nude mice were killed by cervical dislocation, and the tumor samples were weighed. Various relevant indicators were collected and measured. In this study, the humane endpoints we set were mainly based on the animals’ health conditions and tumor growth. If nude mice experience severe weight loss (more than 20% of the initial weight), extreme emaciation, inability to move normally (such as being unable to eat, drink or move independently), tumor ulceration accompanied by signs of infection, or symptoms such as dyspnea that seriously affect the quality of life, we will euthanize the animals. However, in this study, our experimental endpoint was 28 days after cell injection, and during this process, there was no situation requiring premature euthanasia. All animal experimental protocols were approved by the Ethics Review Committee of Animal Experiments, Kunming Medical University (No. kmmu2021594). All methods were performed in accordance with the relevant guidelines and regulations and the ARRIVE guidelines. During the review process, the Institutional Animal Ethics Committee has comprehensively evaluated our experimental protocol, including aspects such as the experimental period, animal welfare protection measures, and the expected mortality rate.

### 2.3 CCK-8 detection of cell viability

BEAS-2B, H460, and H1299 cells (5×10^3^ cells/well) were inoculated into 96-well plates and cultured at 37 °C in a 5% CO_2_ incubator for 24 hours. The cells in each group were treated according to their groupings, and 10 cells were added to each well after treatment with 10 μL of CCK-8 reagent (C0037, Beyotime, China). The absorbance value of each well was measured using an enzyme-linked immunosorbent assay at 450 nm.

### 2.4 Cell cloning

H460 and H1299 cells were divided into groups of 1 cell per well, and 1×10^3^ cells were inoculated at a density of 6-well plates and then continuously cultured for 2 weeks to form colonies. Then, 5 mL of 4% paraformaldehyde was added to each well, and the mixture was incubated for 15 minutes. The colonies were subsequently fixed with methanol and stained with a Giemsa staining kit for 15 minutes. After washing the cells with tap water, photos were taken and observed. The experiment was repeated 3 times.

### 2.5 Transwell detection of migration

The cells in the H460 and H1299 groups were adjusted to 1×10^5^ cells/mL with serum-free RPMI-1640 medium, after which 200 μL of cell suspension was added to the upper chamber of a Transwell chamber, and 600 μL of RPMI-1640 medium containing 10% fetal bovine serum was added to the lower chamber of the 24-well plate. After 24 hours of cultivation, the cells in the lower chamber were stained with crystal violet (Solarbio, China), and the number of cells at fixed positions in each well was observed under an inverted microscope (CKX53, OLYMPUS, Japan). Three fields of view were selected for counting and photography.

### 2.6 Transwell detection of invasion

H460 and H1299 cells were adjusted to 1×10^5^ cells/mL with serum-free RPMI-1640 medium. Two hundred microliters of cell suspension was added to the Transwell chamber coated with Matrigel, and 600 μL of RPMI-1640 medium containing 10% fetal bovine serum was added to the lower chamber of the 24-well plate. After 24 hours of cultivation, cells in the lower wells were fixed with 4% paraformaldehyde and stained with crystal violet (Solarbio, China). The number of cells at each fixed position in each well was observed under an inverted microscope (CKX53, OLYMPUS, Japan), and 5 fields were selected for counting and photography.

### 2.7 Fe^2+^ detection

The iron content was tested using a kit (Sigma Aldrich, MAK025). Tissue (10 mg) or cells (2 × 10^6^) were rapidly homogenized in 4–10 volumes of iron assay buffer. The samples were centrifuged at 16,000 × g for 10 minutes at 4 °C to remove insoluble material. A total of 25 μL of sample was added to the sample well of the 96-well plate, and the volume was adjusted to 100 μL with assay buffer. Then, 5 μL of iron determination buffer was added to each well, the mixture was stirred evenly, and the mixture was incubated in the dark at 25 °C for 30 minutes. Then, 100 μL of iron probe was added, mixed and incubated in the dark at 25 °C for 60 minutes. Finally, the absorbance was measured at 593 nm.

### 2.8 Western blot detection

Proteins were extracted from H460 and H1299 cells and tumor tissue using RIPA buffer (Sigma–Aldrich, USA) containing 1% protease and phosphatase inhibitors. The protein concentration was determined according to the instructions of the BCA detection kit (Thermo Scientific, USA). The total proteins were separated by SDS‒PAGE. The separated proteins were transferred to a polyvinylidene fluoride (PVDF) membrane (Millipore, USA) and then blocked with 5% skim milk powder at room temperature for 1.5 hours. Primary antibodies against SLC7A11 (1:2000, ab175186, Abcam, UK), GPX4 (1:1000, ab125066, Abcam, UK), FTH1 (1:1000, PA5-27500, Thermo Fisher Scientific, USA), p-PERK (1:1000, PA5-40294, Thermo Fisher Scientific, USA), PERK (1:1000, PA5-99447, Thermo Fisher Scientific, USA), p-eIF2α (1:1000, MA5-15133, Thermo Fisher Scientific, USA), eIF2α (1:1000, AHO0802, Thermo Fisher Scientific, USA), ATF4 (1:1000, ab1371, Abcam, UK), CHOP (1:1000, MA1-250, Thermo Fisher Scientific, USA), and β-actin (1:1000, ab8226, Abcam, UK) were added overnight at 4 °C. The membrane was incubated with a secondary antibody (1:4000, ab97051, Abcam, UK) at room temperature for 1 hour, and the bands were then revealed with an enhanced chemiluminescence (ECL) kit (Millipore, USA). Finally, ImageJ software was used to conduct semiquantitative analysis of the bands.

### 2.9 Flow cytometry detection of calcium ions

After H460 and H1299 cells were grouped and processed, cells in 6-well plates were washed once with Hank’s solution without Ca^2+^ and then incubated with Fluo-3/AM (Life Technologies, USA) reagent at 37 °C for 1 hour. The cells were then washed with Hank’s cold solution without Ca^2+^, resuspended in 400 μL of sample buffer in polystyrene tubes and analyzed using flow cytometry (BD LSRFortessa, USA).

### 2.10 Immunohistochemistry

Paraffin-embedded tissue was dewaxed and rehydrated, and then antigen retrieval was performed in 0.01 M citrate buffer (pH 6.0). The slides were incubated with an antibody against Ki67 (1:200, ab15580, Abcam, UK) at 4 °C overnight, and immunoassays were performed using an HRP-conjugated secondary antibody and a DAB chromogenic agent. The results were photographed and observed under a fluorescence microscope (400857, Nikon, Japan).

### 2.11 Statistical analysis

The data were analyzed using GraphPad Prism version 8 software (GraphPad, USA). All the experiments were repeated at least 3 times. Student’s *t* test was used to compare two groups. Multiple group comparisons were performed using one-way or two-way analysis of variance (ANOVA), followed by Tukey’s post hoc test. P<0.05 was considered statistically significant.

## 3 Results

### 3.1 EC inhibits tumor formation in vivo and promotes ferroptosis

**Fig 1:**
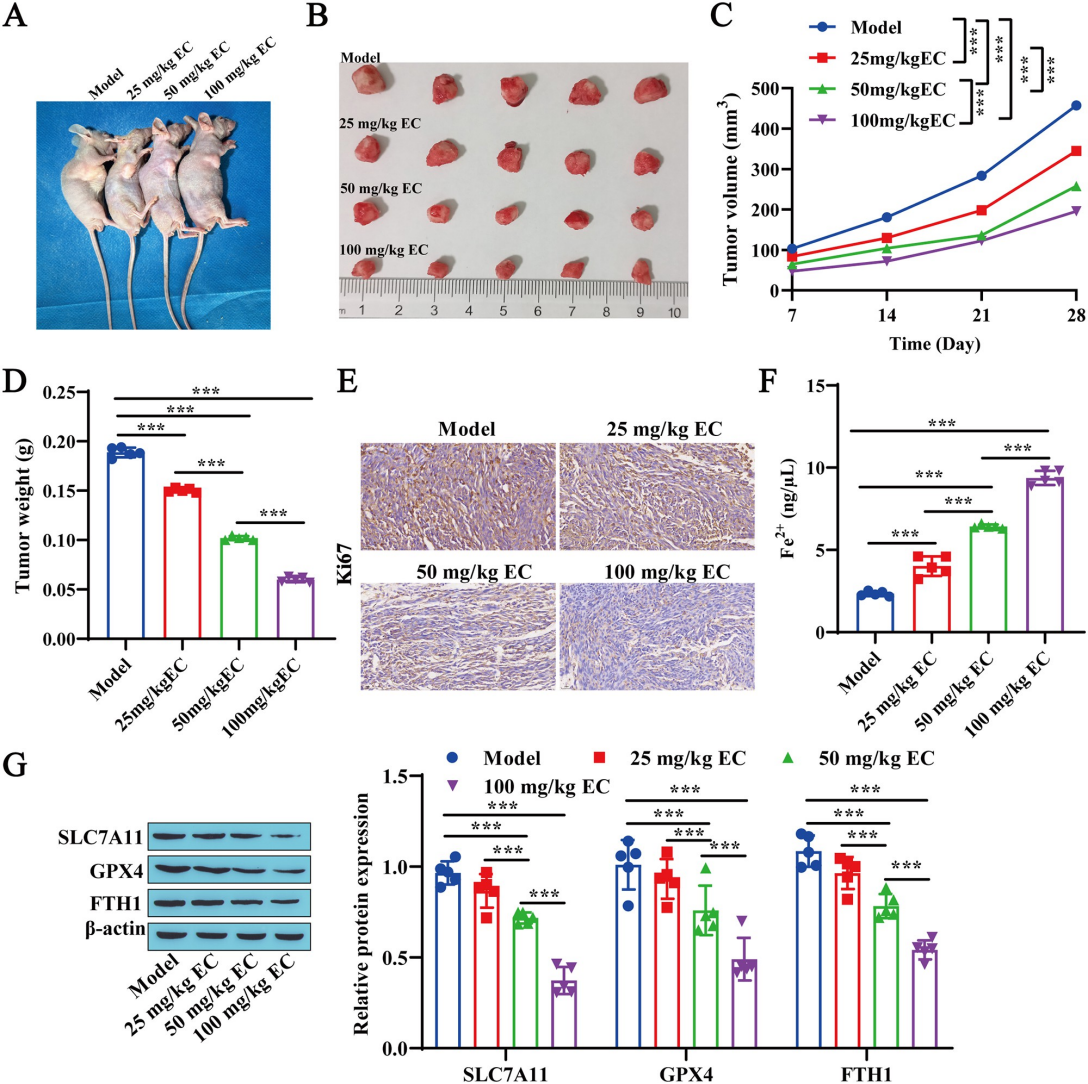
ECs inhibit tumor formation in vivo and promote ferroptosis. A: Representative external images of the tumor-bearing nude mice on the 28^th^ day. B: Tumor size. C: Tumor volume. D: Tumor weight. E: Immunohistochemical detection of Ki-67 expression. F: Detection of Fe^2+^ content using a reagent kit. G: Western blotting was performed to detect the expression levels of the ferroptosis-related proteins SLC7A11, GPX4, and FTH1. *** P<0.001.

We first tested the impact of EC on lung cancer progression and ferroptosis by constructing a nude mouse model of lung cancer. First, the effect of EC treatment on tumor formation was tested. Compared with those of the lung cancer model group, the size, volume, and weight of the tumors decreased with increasing EC concentration (Fig 1A–1D). The immunohistochemical results revealed that, compared with that in the model group, Ki-67 expression decreased with increasing EC concentration (Fig 1E). However, the concentration of Fe^2+^ increased with increasing EC concentration (Fig 1F). Finally, Western blot analysis revealed that, compared with those in the model group, the expression levels of the ferroptosis-related proteins SLC7A11, GPX4, and FTH1 decreased with increasing EC concentration (Fig 1G). These results indicate that EC treatment can inhibit the growth of lung cancer in nude mice and promote ferroptosis.

### 3.2 EC inhibits the proliferation, migration, and invasion of lung cancer cells, inducing ferroptosis

**Fig 2:**
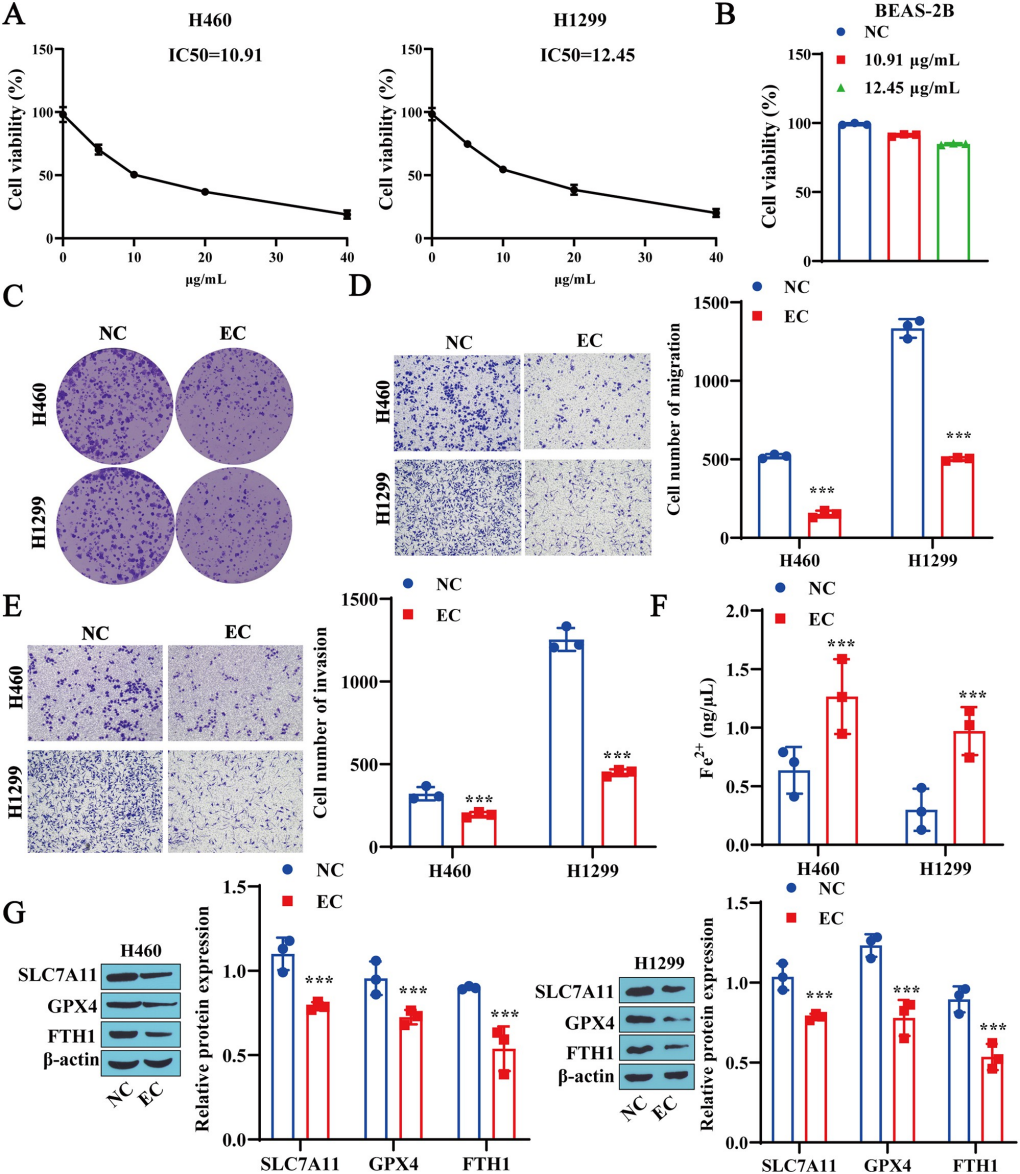
EC inhibits the proliferation, migration, and invasion of lung cancer cells, inducing ferroptosis. A: CCK-8 detection of lung cancer cell viability. B: CCK-8 detection of BEAS-2B cell viability. C: Colony formation assay for detecting lung cancer cell proliferation. D: Transwell assay for detecting lung cancer cell migration. E: Transwell assay for detecting lung cancer cell invasion. F: Detection of Fe^2+^ content in lung cancer cells using a reagent kit. G: Western blotting was used to detect the expression levels of the ferroptosis-related proteins SLC7A11, GPX4, and FTH1 in lung cancer cells. ** P<0.01, * * * P<0.001.

Next, the effects of EC on the malignant biological behavior and ferroptosis of lung cancer cells were tested. First, H460 and H1299 cells were treated with different concentrations of EC, and the IC50 values of EC in H460 and H1299 cells were 10.91 μg/mL and 12.45 μg/mL, respectively (Fig 2A). Therefore, in subsequent experiments, EC concentrations of 10.91 μg/mL and 12.45 μg/mL were selected to treat H460 and H1299 cells, respectively. To exclude the toxicity of EC on human normal pulmonary bronchial epithelial cells, we also treated BEAS-2B cells with the same concentration of EC and found that EC had no significant effect on the viability of these cells (Fig 2B). The effect of EC on the growth of lung cancer cells was subsequently tested. Compared with the NC group, EC treatment significantly inhibited the proliferation, migration, and invasion of H460 and H1299 cells (Fig 2C–2E). Finally, the effect of EC on ferroptosis in lung cancer cells was tested, and the results revealed that EC treatment significantly increased the Fe^2+^ concentrations in H460 and H1299 cells (Fig 2F) while inhibiting the expression of the ferroptosis-related proteins SLC7A11, GPX4, and FTH1 (Fig 2G). These results indicate that EC treatment significantly inhibits the malignant biological behavior of lung cancer cells while promoting ferroptosis.

### 3.3 A ferroptosis inhibitor (Fer-1) reduces EC-induced ferroptosis in lung cancer cells

**Fig 3:**
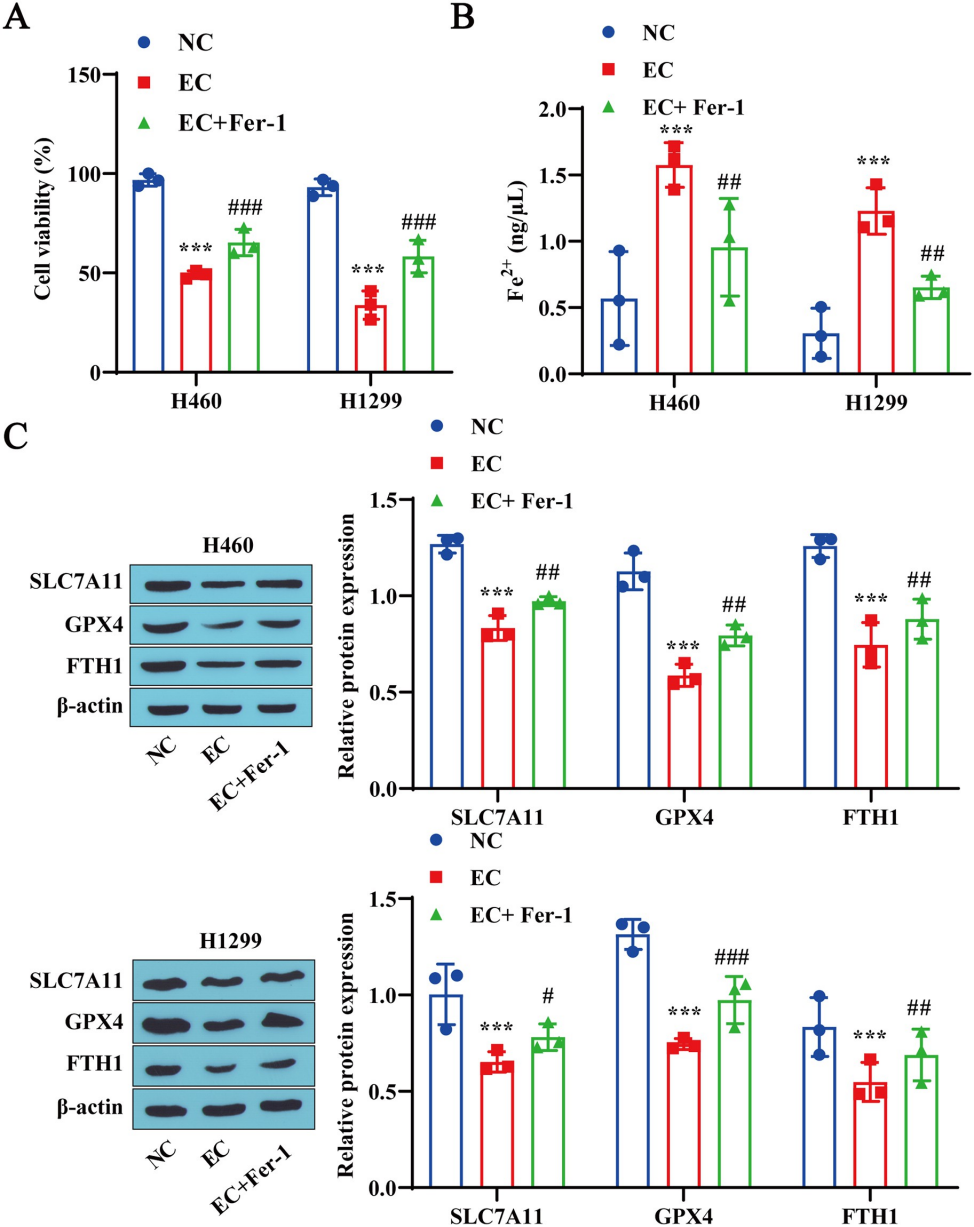
A ferroptosis inhibitor (Fer-1) alleviates EC-induced ferroptosis in lung cancer cells. A: CCK-8 detection of lung cancer cell viability. B: Detection of Fe^2+^ contents in lung cancer cells using a reagent kit. C: Western blotting was used to detect the expression levels of the ferroptosis-related proteins SLC7A11, GPX4, and FTH1 in lung cancer cells. *** P<0.001 vs. NC# P<0.05, # # P<0.01, # # # P<0001 vs. EC.

To further validate the role of ferroptosis in EC-induced cell death, we investigated the effect of Fer-1 on EC-induced lung cancer cell death. We found that Fer-1 treatment weakened the inhibitory effect of EC on H460 and H1299 cell viability and promoted cell viability (Fig 3A). In addition, compared with the EC group, Fer-1 treatment reduced the Fe^2+^ concentrations in H460 and H1299 cells (Fig 3B) while promoting the expression of the ferroptosis-related proteins SLC7A11, GPX4, and FTH1 (Fig 3C). These results suggest that Fer-1 weakens EC-induced ferroptosis in lung cancer cells.

### 3.4 A ferroptosis inducer (erastin) promotes EC-induced ferroptosis in lung cancer cells

**Fig 4:**
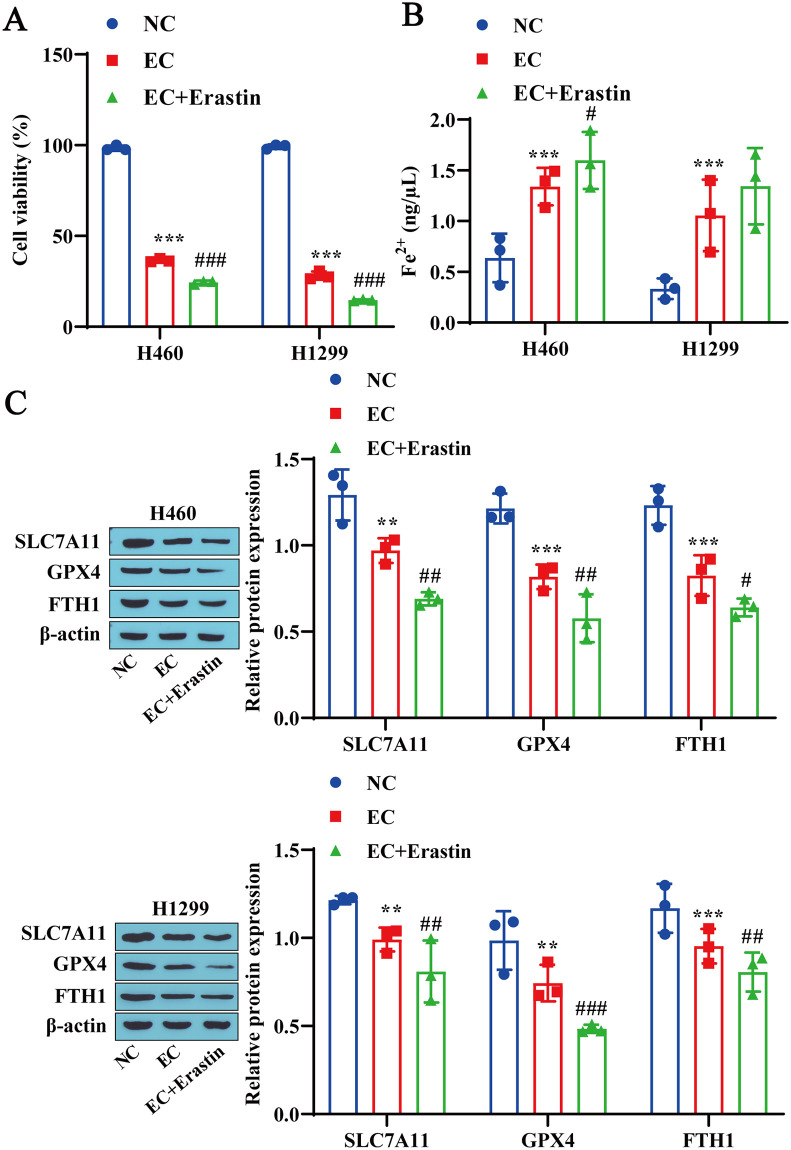
A ferroptosis inducer (erastin) promotes EC-induced ferroptosis in lung cancer cells. A: CCK-8 detection of lung cancer cell viability. B: Detection of Fe^2+^ contents in lung cancer cells using a reagent kit. C: Western blotting was used to detect the expression levels of the ferroptosis-related proteins SLC7A11, GPX4, and FTH1 in lung cancer cells. ** P<0.01, * * * P<0.001 vs. NC# P<0.05, # # P<0.01, # # # P<0001 vs. EC.

The effect of erastin on EC-induced lung cancer cell death was subsequently explored. We found that erastin treatment further intensified the inhibitory effect of EC on the activity of H460 and H1299 cells (Fig 4A). Moreover, compared with the EC group, erastin treatment increased the Fe^2+^ concentrations in H460 and H1299 cells (Fig 4B) while downregulating the expression levels of the ferroptosis-related proteins SLC7A11, GPX4, and FTH1 (Fig 4C). These results suggest that erastin exacerbates EC-induced ferroptosis in lung cancer cells.

### 3.5 ER stress participates in EC-induced ferroptosis in lung cancer cells

**Fig 5:**
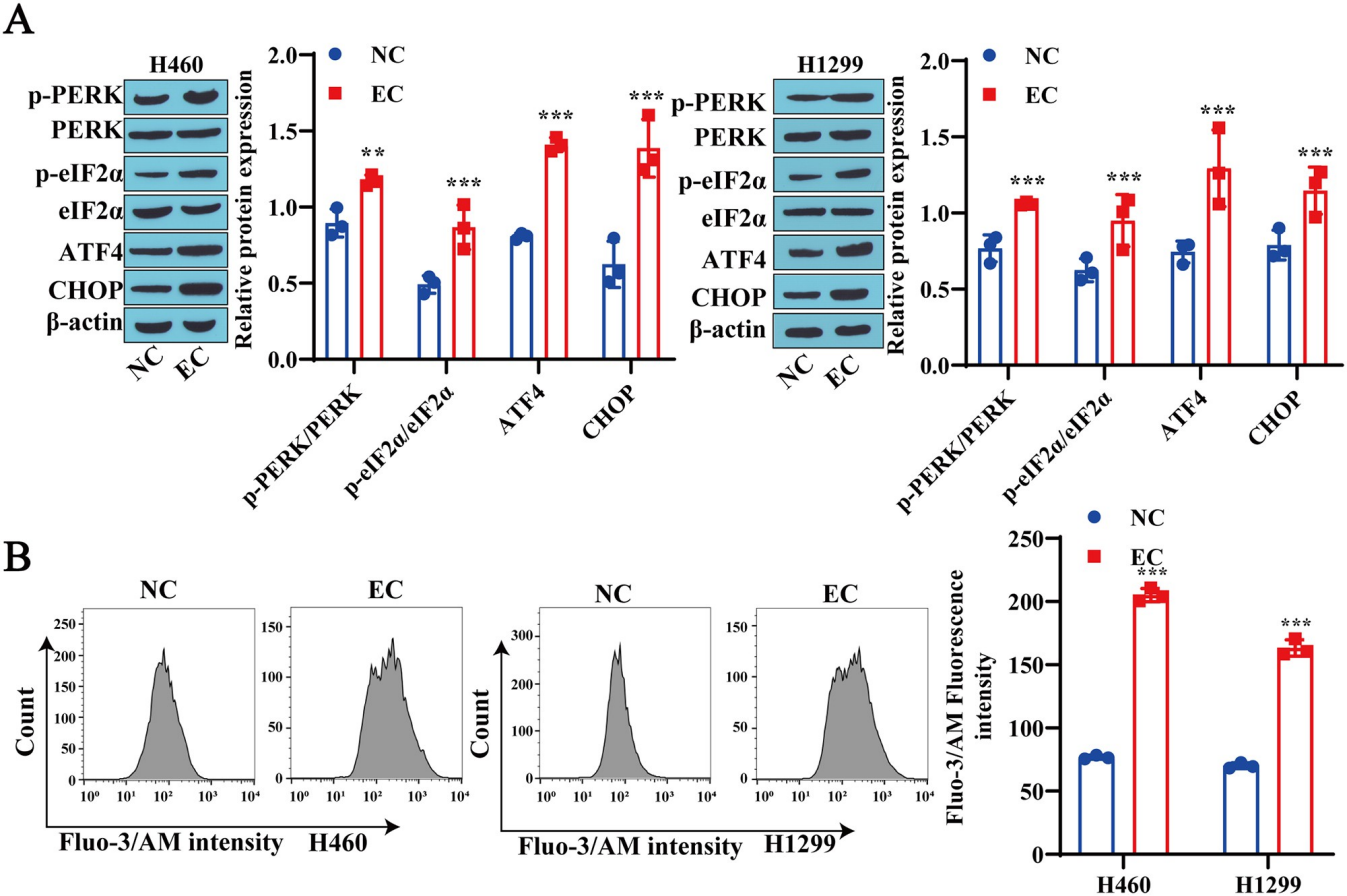
ER stress is involved in EC-induced ferroptosis in lung cancer cells. A: Western blotting was used to detect the expression levels of PERK/eIF2α/ATF4 signaling pathway-related proteins and CHOP in lung cancer cells. B: Flow cytometry was used to measure the Ca^2+^ levels in lung cancer cells. ** P<0.01, * * * P<0.001.

Research has shown that PERK/eIF2α/ER stress mediated by the ATF4 signaling pathway is involved in regulating the process of ferroptosis [21]. Therefore, we hypothesize that ER stress is involved in EC-induced ferroptosis. Compared with those in the NC group, the protein expression levels of p-PERK, p-eIF2α, ATF4 and CHOP in the EC group were significantly increased (Fig 5A). In addition, we found that EC treatment increased the Ca^2+^ levels in H460 and H1299 cells (Fig 5B). These results indicate that ER stress is involved in EC-induced ferroptosis in lung cancer cells.

### 3.6 Inhibition of ER stress reduces EC-induced ferroptosis in lung cancer cells

**Fig 6:**
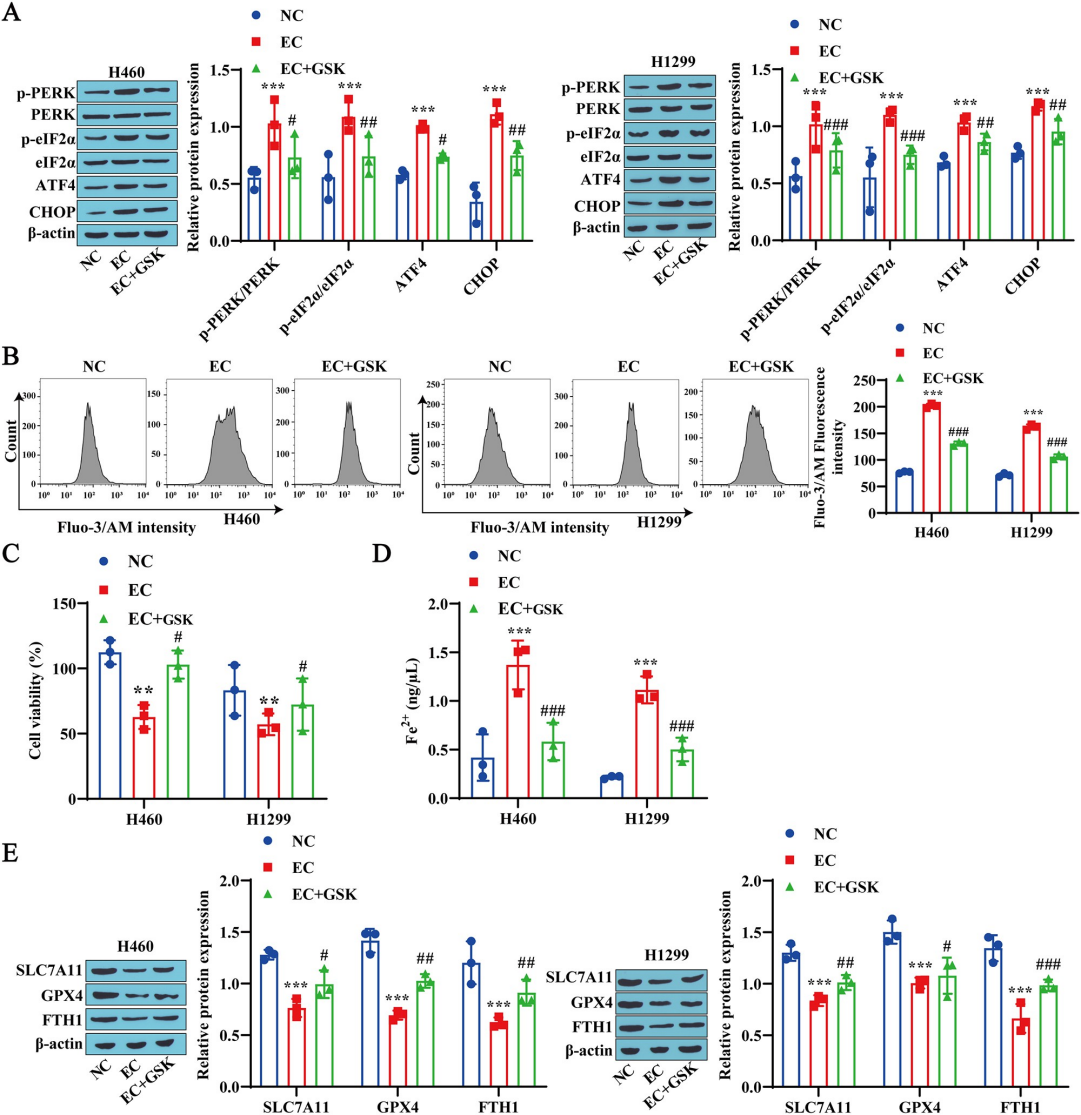
Inhibiting ER stress alleviates EC-induced ferroptosis in lung cancer cells. A: Western blotting was used to detect the expression levels of PERK/eIF2α/ATF4 signaling pathway-related proteins and CHOP in lung cancer cells. B: Flow cytometry was used to measure the Ca^2+^ levels in lung cancer cells. C: CCK-8 detection of lung cancer cell viability. D: Detection of Fe^2+^ content in lung cancer cells using a reagent kit. E: Western blotting was used to detect the expression levels of the ferroptosis-related proteins SLC7A11, GPX4, and FTH1 in lung cancer cells. ** P<0.01, * * * P<0.001 vs. NC# P<0.05, # # P<0.01, # # # P<0001 vs. EC.

To investigate whether ER stress is a cause of EC-induced ferroptosis in lung cancer cells, we treated these cells with the PERK inhibitor GSK before EC treatment. Our results revealed that EC promoted the expression of the ER stress-related proteins p-PERK, p-eIF2α, ATF4 and CHOP in H460 and H1299 cells, whereas GSK treatment inhibited the expression of ER stress-related proteins (Fig 6A). The flow cytometry results revealed that, compared with the EC group, the GSK treatment group presented lower Ca^2+^ levels in H460 and H1299 cells (Fig 6B). Next, we determined the role of ER stress in EC-induced cell death and found that GSK treatment reversed the effects of EC and promoted cell viability (Fig 6C). Compared with the EC group, GSK treatment also reduced the Fe^2+^ concentrations in H460 and H1299 cells (Fig 6D). Finally, we found that GSK treatment promoted the expression of the ferroptosis-related proteins SLC7A11, GPX4, and FTH1 (Fig 6E). These results suggest that the inhibition of ER stress weakens EC-induced ferroptosis in lung cancer cells.

## 4 Discussion

Lung cancer is a malignant tumor caused by various factors, such as smoking, air pollution, and genetic factors [22]. Emerging studies have shown that promoting ferroptosis is an effective method for treating lung cancer [23]. Here, we found that EC is a drug candidate that links ferroptosis with lung cancer progression. The following findings support this conclusion. First, the process of ferroptosis is inhibited in lung cancer. Second, EC can inhibit not only the malignant biological behavior of lung cancer cells in vitro but also tumor formation in vivo while inducing ferroptosis. Third, the process of EC-induced ferroptosis in lung cancer cells is accompanied by the occurrence of ER stress. Finally, we revealed that EC therapy activates the PERK/eIF2α/ATF4 signaling pathway and promotes ER stress, thereby inducing ferroptosis in lung cancer cells and inhibiting the occurrence and development of lung cancer.

Lung cancer has become a major public health problem in China, and the incidence rate has also shown an increasing trend in recent years [24], which has severely affected people’s quality of life. Therefore, understanding the relevant mechanisms of lung cancer occurrence can help in the identification of safe and effective therapeutic drugs. *Fagopyrum dibtrys* (D. Don) Hara is a natural plant with medicinal and edible homology, and its active ingredients have been proven to have excellent antitumor effects [25]. EC, one of the active ingredients of *Fagopyrum dibtrys* (D. Don) Hara, plays important roles in the occurrence and progression of a variety of tumors. For example, Kim et al. [26] reported that EC can inhibit the growth of SW480 human colon cancer cells and induce their apoptosis. In addition, Suganuma et al. [27] reported that EC can promote the apoptosis of PC-9 lung cancer cells and inhibit their growth. Varela et al. [28] reported that EC also inhibits the proliferation of the lung cancer cell line A549 and promotes apoptosis. In our study, we investigated the effect of EC on lung cancer cells and reported that EC treatment could inhibit the proliferation, migration, and invasion of H460 and H1299 cells while inducing ferroptosis. Our research and previous studies have shown that EC has a therapeutic effect on lung cancer, but our study further revealed that the inhibitory effect of EC on the malignant biological behavior of lung cancer cells is achieved by inducing ferroptosis. In addition, our experiments revealed that EC can selectively inhibit lung cancer cells without toxic effects on normal cells (BEAS-2B). We hypothesize that there are significant differences in multiple biological characteristics between cancer cells and normal cells, which makes it possible for ECs to selectively inhibit cancer cells. For example, the overexpression of oncogenes in cancer cells and the inactivation of tumor suppressor genes increase their sensitivity to the action of EC, the metabolic pathways of cancer cells (such as the Warburg effect) differ from those of normal cells, and EC may inhibit cancer cells by interfering with these specific metabolic pathways. In summary, EC may selectively inhibit cancer cells without toxic effects on normal cells through its unique molecular mechanism and cell-specific differences. These findings further support the potential value of EC in cancer treatment.

Ferroptosis is an iron-dependent nonapoptotic form of cell death with unique morphological characteristics and molecular mechanisms. Previous studies have shown that ER stress is involved in inducing ferroptosis [29]. ER stress in solid tumors is caused by imbalances in protein synthesis, folding, secretion, or posttranslational modification and can be triggered by environmental stimuli such as nutritional deficiency, hypoxia, chronic viral infection, oxidative stress, and anticancer drugs [30]. Fang et al. [31] reported that IFN-γ-induced ER stress can promote apoptosis in lung cancer cells. Li et al. [32] reported that pazopanib inhibits the proliferation of small cell lung cancer cells and promotes their apoptosis by inducing ER stress processes. More importantly, studies have shown that ER stress centered on Ca^2+^ bursts can increase ferroptosis in lung cancer, thereby inhibiting its progression [33]. In our study, we also revealed that ER stress is involved in EC-induced ferroptosis in lung cancer cells and that inhibiting ER stress can weaken this process. Studies have also shown that ER stress can lead to the continuous release of stored calcium in the ER into the cytoplasm [34]. Therefore, we also tested the effect of EC treatment on the Ca^2+^ concentration and reported that EC treatment significantly increased the Ca^2+^ concentration in lung cancer cells. These findings indicate that EC therapy induces ferroptosis in lung cancer cells by activating ER stress, thereby inhibiting the malignant biological behavior of these cells.

How do ECs activate ER stress in lung cancer cells? ER stress, a tense biological state in cells, can lead to instability of the intracellular environment. In response to ER stress, cancer cells initiate an evolutionarily conserved signaling process called the unfolded protein response, which is coordinated by three endoplasmic reticulum transmembrane binding sensors: inositol requires kinase enzyme 1 α (IRE1 α), activating transcription factor 6 (ATF6) and protein kinase R-like endoplasmic reticulum kinase (PERK) [35]. Among the three pathways mentioned above, PERK relies on eIF2α phosphorylation to reduce protein synthesis by inhibiting 5’ cap-dependent translation while selectively increasing the cap-independent translation of ATF4, thereby activating factor C/EBP homologous protein (CHOP)-mediated cell apoptosis transcription [32]. Previous studies have shown that activating the PERK–eIF2α–ATF4 signaling pathway can promote the occurrence of ER stress [36]. Moreover, Zhao et al. [21] reported that cadmium can increase ER stress by activating the PERK–eIF2α–ATF4–CHOP pathway, thus promoting ferroptosis in renal tubular epithelial cells. These results indicate that the PERK–eIF2α–ATF4 pathway is the main pathway involved in activating ER stress. Here, we found that EC therapy upregulated the expression levels of p-PERK, p-eIF2α, ATF4, and CHOP, whereas the addition of PERK inhibitors inhibited EC-induced ER stress. These results indicate that EC treatment activates the PERK–eIF2α–ATF4 signaling pathway to promote ER stress in lung cancer cells.

## 5 Conclusions

**Fig 7:**
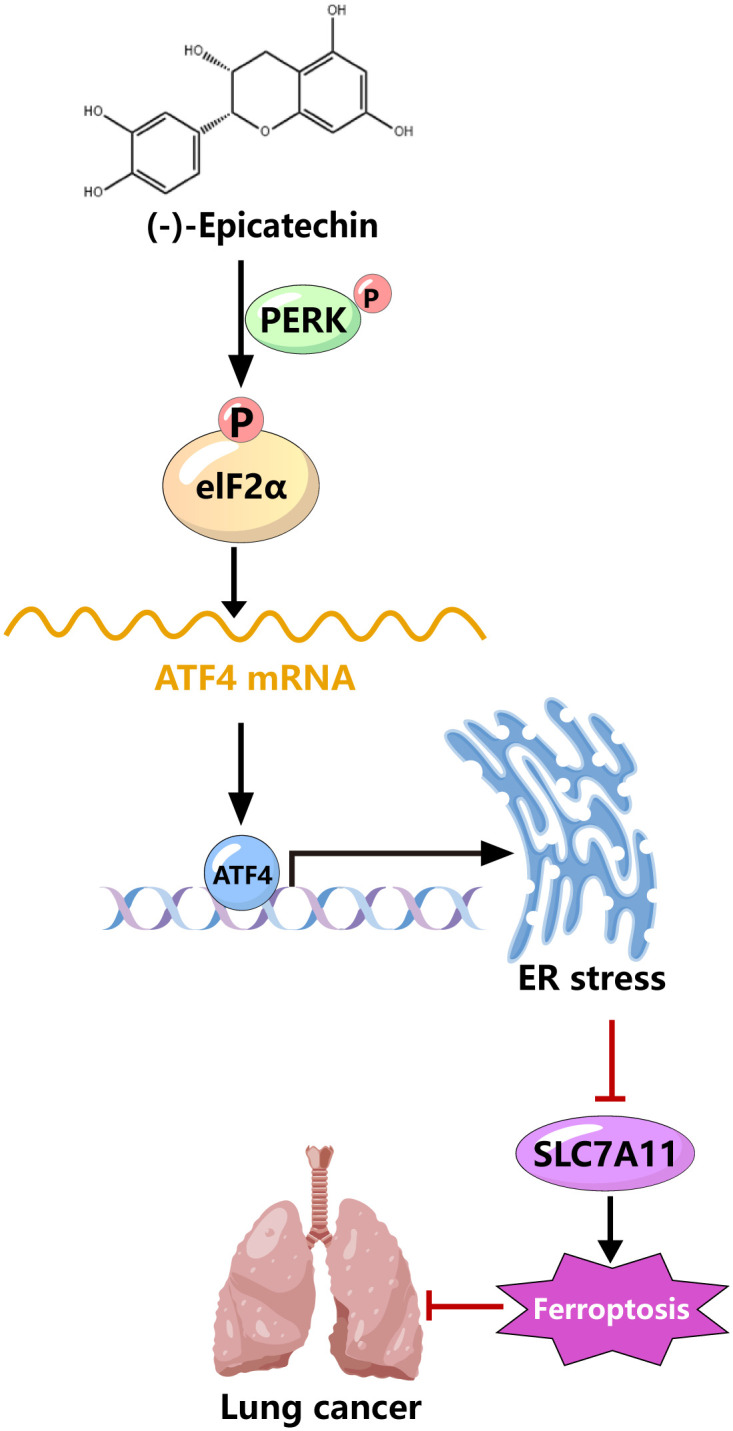
Mechanistic diagram of EC that mitigates lung cancer progression by activating ferroptosis in lung cancer cells.

In conclusion, we found that EC treatment increases ER stress by activating the PERK–eIF2α–ATF4 signaling pathway, thereby promoting iron-related death in lung cancer cells and alleviating lung cancer progression (Fig 7). Our findings suggest that EC has potential as a novel drug for the treatment of lung cancer. However, this study has certain limitations. First, we did not explore the specific mechanism by which ER stress regulates ferroptosis, but based on relevant literature, we speculate that ER stress regulates iron ion levels through calcium release, thereby regulating ferroptosis. This possibility requires further exploration in the future. Second, we detected this phenomenon only through cell and animal experiments; however, clinical trials are still lacking. To apply EC in the treatment of lung cancer, further research is needed in the future. Although EC may regulate other signaling pathways, we demonstrated that the therapeutic effect of EC on lung cancer mainly lies in promoting the iron-mediated death of lung cancer cells.
