# Correction: (−)-Epicatechin regulates endoplasmic reticulum stress and promotes ferroptosis in lung cancer cells via the PERK/eIF2α/ATF4 signaling pathway

**DOI:** 10.1371/journal.pone.0320174

**Published:** 2025-03-10

**Authors:** Zengbo Lv, Peiwan Liu, Yingyu Yang, Jianhua Ji, Anao Wu, Wensheng Huang, Liqiong Zhang, Zhijun Zhang, Yunkui Yang, Wenhui Li, Meifang Huang

The funding statement for this paper is incorrect.

The correct funding statement is as follows: This work was supported by the Science and Technology Department of Yunnan Province-Joint special project general program of Kunming Medical University [202001AY070001-276]; National Natural Science Foundation of China [82160470]; National Natural Science Foundation of China [82060558]; Key Project of Basic Research of Yunnan Province [202001AS070011]; ‘Famous Doctor’ Special Project of Yunnan Province High-level Talents Training Support Plan [YNWR-MY-2020-016]; Science and Technology Department of Yunnan Province- Kunming Medical University Joint Special Key Project of Applied Basic Research [202301AY070001-019]; Science and Technology Department of Yunnan Province- Kunming Medical University Joint Special Key Project of Applied Basic Research [202401AY070001-013].
